# Population Genetic Structure of Ornate Threadfin Bream (*Nemipterus hexodon*) in Thailand

**DOI:** 10.21315/tlsr2021.32.1.4

**Published:** 2021-03-31

**Authors:** Verakiat Supmee, Apirak Songrak, Juthamas Suppapan, Pradit Sangthong

**Affiliations:** 1 Department of Science, Faculty of Science and Technology, Rajamangala University of Technology Srivijaya, Nakhon Si Thammarat Campus, Nakhon Si Thammarat 80110, Thailand; 2 Department of Fishery Technology, Faculty of Science and Fisheries Technology, Rajamangala University of Technology Srivijaya, Trang Campus, Trang 92150, Thailand; 3 Faculty of Education, Nakhon Si Thammarat Rajabhat University, Nakhon Si Thammarat 80280, Thailand; 4 Department of Genetics, Faculty of Science, Kasetsart University, Bangkok 10900, Thailand

**Keywords:** *COI* Gene, Demersal Fish, Genetic Diversity, Mitochondrial DNA, Thailand

## Abstract

Ornate threadfin bream (*Nemipterus hexodon*) is an economically important fishery species in Southeast Asia. In Thailand, *N. hexodon* decreased dramatically due to overexploitation for commercial purposes. To construct an effective sustainable management plan, genetic information is necessary. Thus, in our study, the population genetic structure and demographic history of *N. hexodon* were investigated using 419 bp of the mitochondrial DNA sequence in cytochrome oxidase subunit I gene (mtDNA *COI*). A total of 142 samples was collected from nine localities in the Gulf of Thailand (Chonburi, Samut Songkhram, Surat Thani, Nakhon Si Thammarat, Songkhla), and the Andaman Sea (Satun, Trang, Krabi, Phang Nga). Fourteen polymorphic sites defined 18 haplotypes, revealing a high haplotype diversity and low nucleotide diversity among nine localities. The analysis of molecular variance (AMOVA) analysis, pairwise *F_ST_*, and minimum spanning network result revealed that the genetic structure of *N. hexodon* was separated into two populations: the Gulf of Thailand and the Andaman Sea population. The genetic structure of *N. hexodon* can be explained by a disruption of gene flow from the geographic barrier and the Pleistocene isolation of the marine basin hypothesis. Neutrality tests, Bayesian skyline analysis, mismatch distribution, and the estimated values of population growth suggested that *N. hexodon* had experienced a population expansion. The genetic information would certainly help us gain insight into the population genetic structure of *N. hexodon* living on the coast of Thailand.

**Highlights**

- The population genetic structure of *Nemipterus hexodon* living along the Thailand coast was separated into the Gulf of Thailand and the Andaman Sea populations.
- The management of the *N. hexodon* population along the Thailand coast should be managed as two-unit programs into the Gulf of Thailand and the Andaman Sea populations.
- The multiple demographic history tests indicated that the *N. hexodon* population had experienced population expansion during the Pleistocene glaciation period.

## INTRODUCTION

Ornate threadfin bream (*Nemipterus hexodon*) is a demersal fish, widely distributed in the Indian Ocean to the Pacific Ocean. It is a principal catch species in the trawl fishing in Southeast Asia (Russell *et al.* 2016; Stobutzki *et al.* 2006). The meat of *N. hexodon* has a white colour and it has better gelling than the red-fleshed fish (Hastuti *et al.* 2017). As a result, in Thailand, this species is widely used for surimi production (Nalinanon *et al.* 2011). Approximately 52,000 tons/year of *N. hexodon* is consumed. The annual economic value is approximately 1,560 million baht. There was an apparent increase in the exploitation rate of *N. hexodon* from 2011 to 2013, and the amount of *N. hexodon* reduced sharply from 2014 to 2015 owing to overexploitation for commercial purposes (Fishery Statistics Analysis and Research Group 2017).

Most species of marine fish spend part of their several free-moving stages in the life cycle to open waters and moving by sea current, promoting gene flow between populations (Uthicke & Benzie 2003). However, some factors including a geographic barrier could limit gene flow between the populations and evolved independently of genetic structure. In Thailand, *N. hexodon* is mainly found along the coast of Thailand. Thailand’s coast is divided into two seas (the Gulf of Thailand and the Andaman Sea) by geographic barriers from the Thai-Malaysian peninsula.

This geographic barrier limited gene flow of marine animals that lived between two areas (Held *et al*. 2011). Strong genetic differences in the Thailand Sea have been reported between the Gulf of Thailand and the Andaman Sea population of a variety of marine organisms, i.e., *Varuna litterata* (Suppapan *et al*. 2017), *Portunus pelagicus* (Klinbunga *et al*. 2010), *Hippocampus kuda* (Panithanarak *et al.* 2010), *Epinephelus coioides* (Antoro *et al.* 2006), and *Penaeus merguiensis* (Wanna *et al*. 2004). Hence, geographic barriers may affect the genetic structure of *N. hexodon* living between the Gulf of Thailand and the Andaman Sea.

The mitochondrial genome is an exclusively relatively rapid evolutionary rate, maternally inherited-a lack of recombination, and easy to amplification (Avise 2000). The cytochrome oxidase subunit I gene (mtDNA *COI*) is the one of the most frequently used mitochondrial genes and has been widely used to investigate the population genetic structure of marine organisms (Xu *et al*. 2014; Sun *et al*. 2011). The population genetic structure of several marine fish has been examined with mtDNA *COI* such as *Sardinella longiceps* (Sukumaran *et al*. 2016), *Larimichthys polyactis* (Zhang *et al.* 2017) and *Lateolabrax maculates* (Wang *et al*. 2017). In our study, we used information from the mtDNA *COI* sequence to discuss the following questions: (1) to examine genetic variation and genetic structure of *N. hexodon*; and (2) to investigate the demographic history of *N. hexodon*. The genetic information would help design the management of *N. hexodon* in Thailand.

## MATERIALS AND METHODS

### Sample Collection, DNA Extraction, Amplification and Sequencing

**Figure 1:**
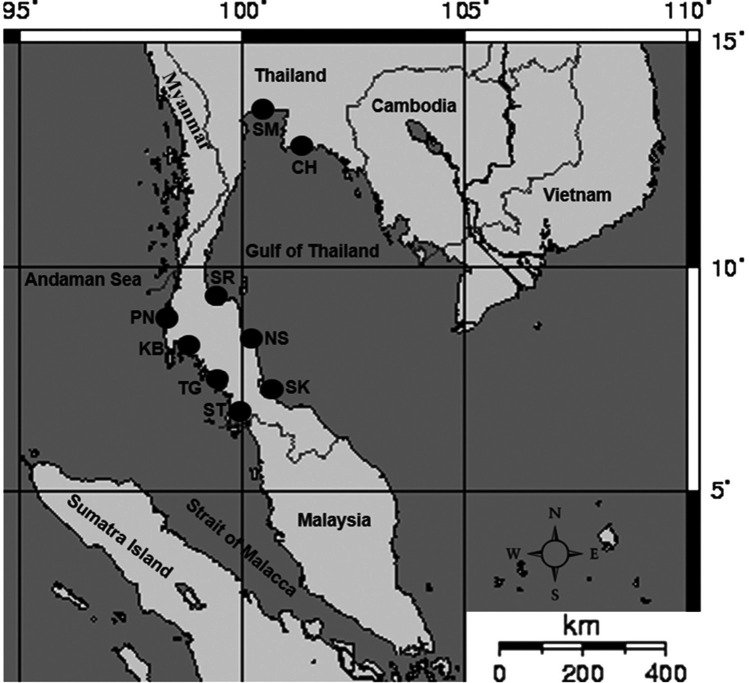
Sampling areas of the *N. hexodon* from the Thailand coast: the Gulf of Thailand; Chonburi (CH), Samut Songkhram (SM), Surat Thani (SR), Nakhon Si Thammarat (NS) and Songkhla (SK), and: the Andaman Sea; Satun (ST), Trang (TG), Krabi (KB) and Phang Nga (PN) (*Source*: Wikimedia Common; https://commons.wikimedia.org/w/index.php?title=File:Straits_of_Malacca.png&oldid=471830265)

The total of 142 fish were caught between November 2014 and July 2015 from the Gulf of Thailand coast including Chonburi (CH), Samut Songkhram (SM), Surat Thani (SR), Nakhon Si Thammarat (NS) and Songkhla (SK), and the Andaman Sea coast including Satun (ST), Trang (TG), Krabi (KB) and Phang Nga (PN) (Table 1, Fig. 1). Samples were preserved on ice, transferred to a laboratory, and stored at −20°C until required. All samples were strictly identified according to the Food and Agriculture Organisation (FAO) description of this species (Russell 1990) before DNA extraction. Total genomic DNA was extracted from *N. hexodon* muscle tissue using a Tissue Genomic DNA Extraction Mini Kit (FAVORGEN, Biotech Corp., Taiwan) according to the manufacturer’s protocol. Based on the mtDNA *COI* sequence database of *N. hexodon* from the National Centre for Biotechnology Information (Accession number: KY362820.1, Hung *et al.* 2017), primers were designed to amplify a 419 bp of mtDNA *COI* gene: NH_COI_H1 5′ CCT TTA TCT CTT ATT TGG TGC C 3′ and NH_COI_L1 5′ GAA GAG ATG TTG ATA AAG AAT GGG 3′.

Polymerase chain reaction (PCR) was prepared in a final volume of 50 μL. The reaction mixture consisted of 2.5 μL of DNA template (50 ng–100 ng), 5 μL of 10X *Taq* buffer, 7.5 μL of 25 mM MgCl_2_, 4 μL of 2 mM dNTPs mix, each 2 μL of 10 μM forward and reverse primers, 0.5 μL of 2.5 unit *Taq* DNA polymerase (ThermoScientific, USA), and 26.5 μL of ultrapure water. The reaction mixtures were amplified in a thermocycler (Major Cycler, Cycle, Taiwan). PCR cycling profile consisted of first denaturation at 94°C for 4 min, followed by 35 cycles of 94°C for 40 s, 51°C for 1 min, 72°C for 1 min, and a final extension at 72°C for 10 min. All PCR products were checked with the correct size on 1% agarose gels (1×TAE), cleaned using a Gel/PCR Purification Mini Kit (FAVORGEN, Biotech Corp.) and sequenced (1st Base Laboratory, Apical Scientific, Malaysia).

### Data Analysis

#### Genetic diversity

Multiple sequence alignments were implemented by using ClustalW version 1.83 (Thompson *et al.* 1994). Ambiguous positions of the aligned sequences were adjusted manually. The polymorphic sites, nucleotide diversity (*π*) (Nei & Tajima 1981) and haplotype diversity (*h*) (Nei 1987), were calculated using DnaSP version 5.00 (Librado & Rozas 2009).

#### Population genetic structure

An analysis of molecular variance (AMOVA) was performed with ARLEQUIN version 3.5 (Excoffier & Lischer 2010) to investigate the population genetic structure. A hierarchical AMOVA was used to estimate the relative contribution of molecular variance: (1) among groups of populations relative to the whole species (*Φ_CT_*), (2) among populations relative to a regional group of populations (*Φ_SC_*), and (3) among populations (*Φ_ST_*: correlation of haplotype within populations relative to those from the whole species). The population genetic structure of *N. hexodon* was investigated under four groups. Firstly, the *N. hexodon* specimens were separated into nine populations according to the sampling areas (CH, SM, SR, NS, ST, SK, TG, KB, PN). Secondly, they were determined according to the two geographic regions: The Gulf of Thailand (CH, SM, SR, NS, SK) and the Andaman Sea (ST, TG, KB, PN). Thirdly, they were determined according to the two geographic regions: the lower Gulf of Thailand (SR, NS, SK) and the upper Gulf of Thailand (CH, SM). Fourthly, they were determined according to the two geographic regions: the lower Andaman Sea (ST, TG) and the upper Andaman Sea (KB, PN). Genetic distances between all possible pairwise *F_ST_* combinations of populations were estimated within the region and between regions to strengthen the genetic structuring hypothesis. The significance of the pairwise differentiation was tested with 10 000 permutations. A minimum spanning network (MSN) was constructed and drawn by hand to show the relationship among haplotypes using ARLEQUIN version 3.5 (Excoffier & Lischer 2010).

#### Demographic history

The demographic history of *N. hexodon* from all localities across the Thailand coast was examined using four independent approaches. Firstly, Tajima’s D-test (Tajima 1989) and Fu’s Fs test (Fu 1997) were performed in ARLEQUIN version 3.5 (Excoffier & Lischer 2010) using 10 000 replicates, to test for deviations from neutral molecular evolution. Secondly, Bayesian skyline analysis was calculated by using BEAST/BEAUTi version 1.7.2 (Drummond *et al*. 2012) to infer the change of the effective population size (*Ne*) with time. In our analysis, the best substitution models of mtDNA *COI* were recommended by jModelTest 2.1.7 (Darriba *et al*. 2012). Based on Di Battista *et al*. (2013), the evolutionary rate of mtDNA *COI* was adjusted to 2.0% per million years. The analysis was conducted with 20 million steps in a Markov chain Monte Carlo (MCMC) simulation with a relaxed molecular clock model. The result was generated by Tracer version 1.6 (Rambaut *et al*. 2014). Thirdly, mismatch distributions (Rogers & Harpending 1992) were estimated to check that a population had undergone a sudden population expansion (Rogers 1995). Harpending raggedness index (Harpending 1994) and the sum of squared deviations (SSD) of the goodness-of-fittest (Schneider & Excoffier 1999) were tested the fit between observed and expected mismatch distribution with 10 000 bootstrap replicates, as implemented in ARLEQUIN version 3.5 (Excoffier & Lischer 2010). Fourthly, two parameters: θ_0_ (population size before expansion, θ_0_ = 2N_0_μ) and θ_1_ (population size after expansion, θ_1_ = 2N_1_μ) (Rogers 1995) of the mismatch distribution were calculated.

## RESULTS

### Genetic Diversity

The alignment results showed that out of 419 aligned sites, 405 were monomorphic and 14 were polymorphic. Among the 14 polymorphic sites, 3 sites were a singleton, and 11 sites were informative parsimony sites. The samples from the Gulf of Thailand had 9 polymorphic sites and the Andaman Sea had 5 polymorphic sites. The alignment of variable sites is presented in Table 1.

Haplotype diversity value ranged from 0.133 ± 0.112 to 0.792 ± 0.089 in Thailand, 0.582 ± 0.092 to 0.792 ± 0.089 in the Gulf of Thailand, and 0.133 ± 0.112 to 0.614 ± 0.117 in the Andaman Sea. Haplotype diversity of the Gulf Thailand and the Andaman Sea was 0.740 ± 0.035 and 0.468 ± 0.074, respectively (Table 2). Nucleotide diversity value ranged from 0.000 ± 0.000 to 0.003 ± 0.000 in Thailand, 0.001 ± 0.000 to 0.003 ± 0.000 in the Gulf of Thailand, and 0.000 ± 0.000 to 0.001 ± 0.000 in the Andaman Sea (Table 2). Nucleotide diversity of the Gulf Thailand and the Andaman Sea was 0.002 ± 0.000 and 0.001 ± 0.000, respectively. In total, 18 haplotypes were identified. In the Gulf of Thailand, 12 haplotypes were found. Haplotype H07 and H10 were shared by all populations from the Gulf of Thailand. Five haplotypes were specific within the population called “rare haplotype” (Table 3). In the Andaman Sea, 6 haplotypes were found. Haplotype H02 was shared by all populations from the Andaman Sea and one population from the Gulf of Thailand. The Andaman Sea population did not have a rare haplotype (Table 3). Eighteen haplotypes of mtDNA *COI* sequences have been deposited into GenBank; accession numbers MH361216-MH361233. The number of polymorphic sites, number of haplotypes, haplotype diversity (*h*), and nucleotide diversity (*π*) within each population are presented in Table 2.

### Population Genetic Structure

**Figure 2:**
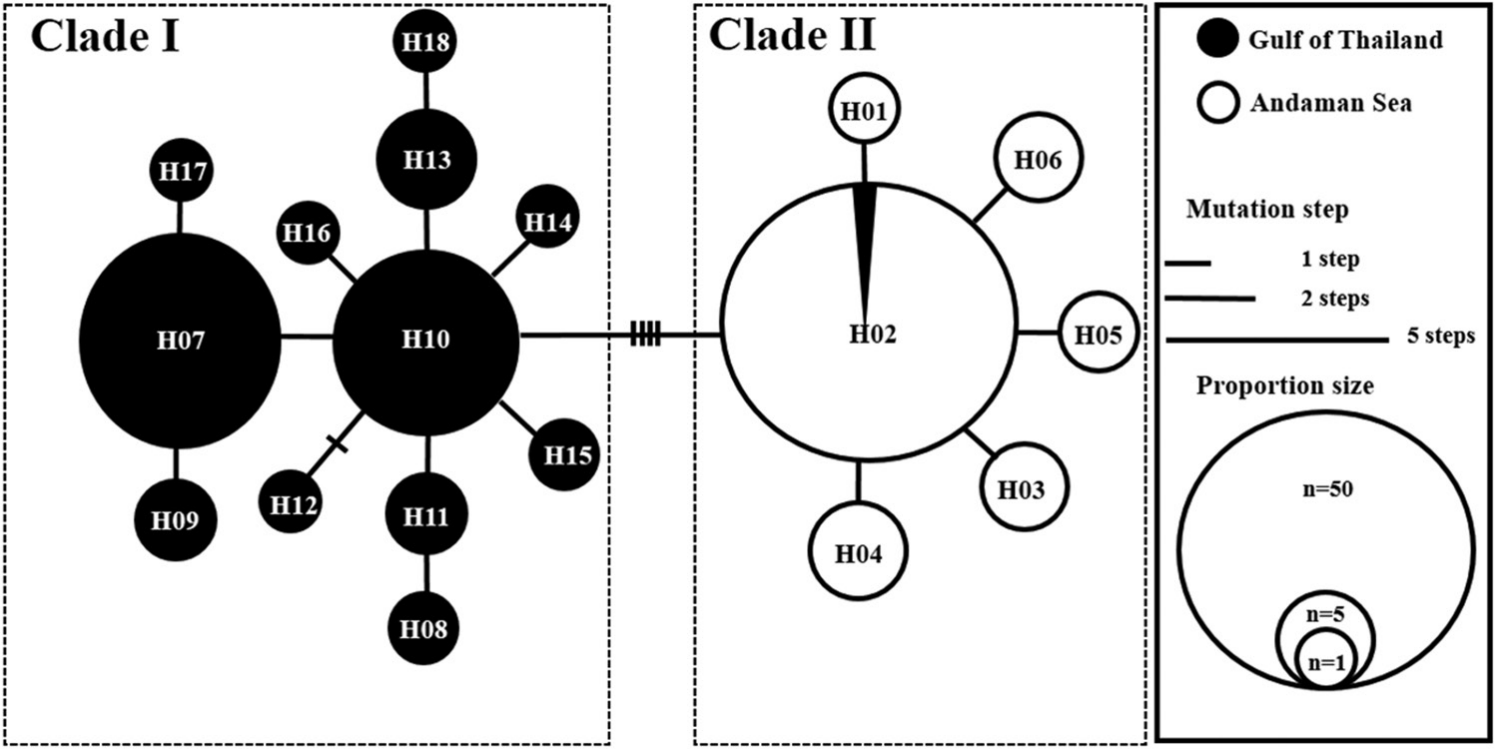
A minimum spanning network of the 18 mitochondrial haplotypes of the *N. hexodon.* The sizes of the circles are proportional to the frequency of haplotypes. The vertical bars on the line indicate the number of mutation step separating two haplotypes. A lack of vertical bars on the line connecting haplotypes indicates that a single mutation step separates two haplotypes.

The population genetic structures of *N. hexodon* collected from the Thailand coast and based on the mtDNA *COI* sequence were determined. The AMOVA result for the overall population showed a highly significant difference (*Φ_ST_* = 0.460, *p* = 0.000), indicating a genetic structuring among *N. hexodon* population in Thailand (Table 4). The hierarchical AMOVA revealed a strong genetic structure between the Gulf of Thailand and the Andaman Sea (*Φ_CT_* = 0.547, *p* = 0.008) (Table 4). In the Gulf of Thailand, the hierarchical AMOVA showed no significant difference between the lower and upper Gulf of Thailand (*Φ_CT_* = 0.133, *p* = 0.098), indicating that the *N. hexodon* population in the Gulf of Thailand was single (Table 4). In the Andaman Sea, the hierarchical AMOVA revealed that the lower Andaman Sea was not significantly different from the upper Andaman Sea (*Φ_CT_* = −0.009, *p* = 0.672), indicating a lack of genetic structure of *N. hexodon* in the Andaman Sea (Table 4). Furthermore, evidence of genetic differentiation between the regions was revealed by pairwise *F_ST_* analysis as shown in Table 5. Every pairwise *F_ST_* of the geographic-based populations showed significant differences for most comparisons between the Gulf of Thailand and the Andaman Sea populations. The haplotype network of *N. hexodon* was divided into two clades from a distinct pattern of geographic structure among 18 haplotypes (Fig. 2). Clade I consisted of all haplotypes from the Gulf of Thailand and a common haplotype was haplotype H10. Clade II consisted of haplotypes from the Andaman Sea in a large proportion and a common haplotype was haplotype H02. These two clades were separated by 5 mutation steps (Fig. 2). The resultant network exhibited star-like patterns surrounding common haplotype H10 and H02.

### Demographic History

**Figure 3:**
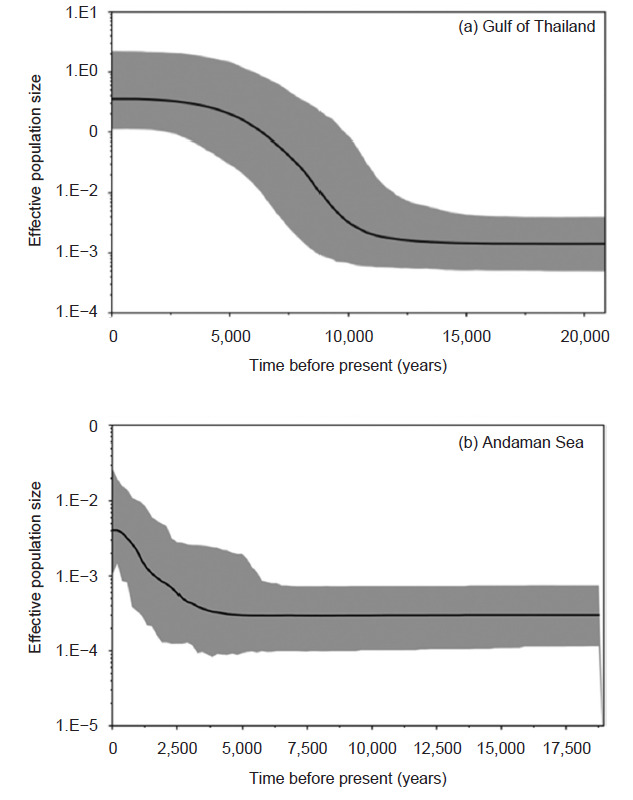
Bayesian skyline plots show the changes in effective population size over time. Central line: median estimation; grey colour: limits of 95% confidence interval.

**Figure 4:**
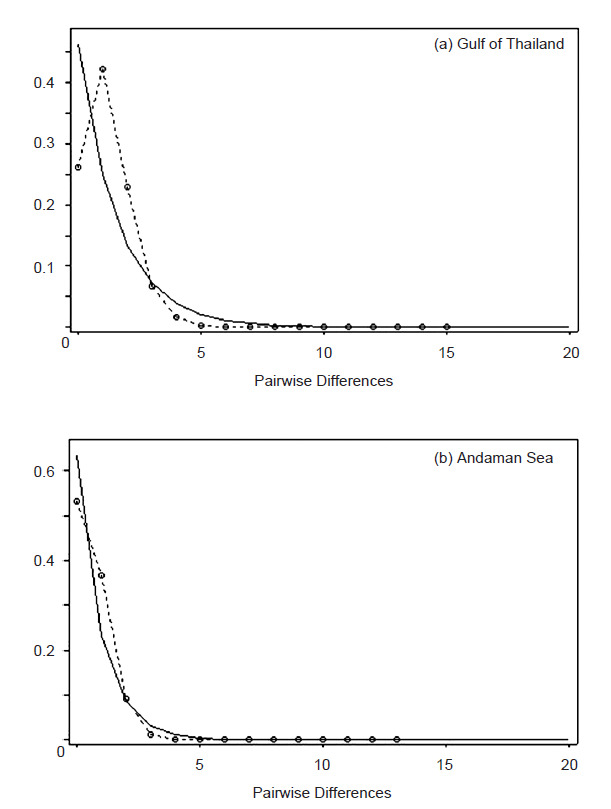
The observed pairwise differences (dotted line) and the expected mismatch distribution (thin line) under sudden population expansion model of *N. hexodon*.

Tajima’s *D* and Fu’s *Fs* showed negative values in every population (Table 6). Tajima’s *D* statistic was in the range −0.067 to −1.159 in Thailand, −0.067 to −0.576 in the Gulf of Thailand, and −0.438 to −1.159 in the Andaman Sea (Table 6). Tajima’s *D* statistic was significantly negative for the Gulf of Thailand (*D* = −1.034, *p* = 0.026) and the Andaman Sea (*D* = −0.941, *p* = 0.037). Fu’s *Fs* statistic was in the range −0.055 to −2.360 in Thailand, −0.055 to −2.360 in the Gulf of Thailand, and −0.648 to −2.095 in the Andaman Sea (Table 6). The Fu’s *Fs* test showed significantly negative values in the Gulf of Thailand (*Fs* = −2.479, *p* = 0.002) and the Andaman Sea (*Fs* = −5.918, *p* = 0.008) (Table 6). The Bayesian skyline analysis was used to estimate the population expansion time and found that population expansion had occurred around 10 000 years ago in the Gulf of Thailand population (Fig. 3a) and 4,000 years ago in the Andaman Sea population (Fig. 3b). Mismatch distribution of the Gulf of Thailand and the Andaman Sea fitted the unimodal distribution (Figs. 4a, 4b). The measurement of SSD from the goodness of fit test, the mismatch distribution observed from the Gulf of Thailand (SSD = 0.001, *p* = 0.154), and the Andaman Sea (SSD = 0.005, *p* = 0.085) did fit a sudden expansion model (Table 6). The raggedness statistic (rg), which measured the smoothness of the mismatch distribution (Harpending 1994) in the Gulf of Thailand (rg = 0.109, *p* = 0.124) and the Andaman Sea (rg = 0.092, *p* = 0.111) was not statistically significant (Table 6). The estimated *θ_1_* was higher than *θ*_0_ for the Gulf of Thailand and the Andaman Sea, indicating the population expansion from a small to a large size (Table 6).

## DISCUSSION

### Genetic Diversity

In our study, the pattern of genetic diversity highly revealed a value of haplotype diversity and a low value of nucleotide diversity. Grant and Bowen (1998) categorised population dynamics into four scenarios from the different combinations of values of haplotype diversity and nucleotide diversity. The second category consists of populations with high haplotype diversity and low nucleotide diversity. The population has experienced rapid expansion after a period of low effective population size. The cause of the population growth is responsible for retaining new mutations and keeping up high haplotype diversity within a population (Ma *et al*. 2010; Rogers & Harpending 1992; Watterson 1984). Besides, the low nucleotide diversity may be purported to a short existence of haplotypes. Hence, newly created haplotypes go extinct after obtaining more base pair differences (Cassone & Boulding 2006). According to Grant and Bowen’s criteria, the variability pattern of genetic diversity in this study indicates that *N. hexodon* has experienced a population expansion. This pattern has been reported as a typical molecular character of marine fish species, such as the Chinese sea bass (*L. maculatus*) (Wang *et al*. 2017), the small yellow croaker (*L. polyacis*) (Zhang *et al*. 2017) and Indian oil sardine (*S. longiceps*) (Sukumaran *et al*. 2016). In our study, the fish population from the Andaman Sea had lower genetic diversity than the Gulf of Thailand population. Therefore, this area should be focused on to aid in determining breeding populations and proper units of management for conservation. For example, to enhance the genetic diversity of *N. hexodon* populations in the Andaman Sea, the regulatory enforcement in capturing and trade must be used to prevent population declines.

### Population Genetic Structure

The AMOVA analysis revealed that the genetic structure of *N. hexodon* was separated into two populations including the Gulf of Thailand and the Andaman Sea population, corresponding well with the geographic regions. Further pairwise *F_ST_* analysis and a minimum spanning network showed the genetic structure between the *N. hexodon* population living in the Gulf of Thailand and the Andaman Sea. The distinct genetic separation between the two populations may be partly explained by gene flow disruption from the geographic barrier caused by the Thai-Malaysian peninsula. Genetic differentiation between these two regions has been observed in various marine species, such as *Haliotis asinina* (Tang *et al*. 2005), *Thenus indicus* (Iamsuwansuk & Denduangboripant 2011), *Amusium pleuronectes* (Mahidol *et al*. 2007), and *H. kuda* (Panithanarak *et al*. 2010). Various studies have also tested the relation between morphological and genetic grouping. Many results have found that the morphology of organisms is different in each population group. On the other hand, a morphological study of some species failed to support the genetic groupings such as *Nothobranchius furzeri* (Bartáková *et al*. 2013) and *Myotomys unisulcatus* (Edwards *et al.* 2011). In our study, there were no differences in the morphology of *N. hexodon* between the Gulf of Thailand and the Andaman Sea. The result showed that there was only one population of *N. hexodon* along the 1,800 km of Gulf of Thailand coastline and the 900 km of the Andaman Sea coastline. There are two main factors to promote gene flow of marine animals in the open sea: a geographic distance and oceanographic current (Chambers *et al*. 2006; Neethling *et al*. 2008). In general, marine species-gametes, planktonic larvae, and adults in open waters-which have a high dispersal ability by spending part of their life cycle are distributed by a marine current. This strategy promoted gene flow among marine species population (Lacson 1992; Ovenden *et al*. 1992; Russo *et al*. 1994; Uthicke & Benzie 2003). The evidence of genetic connectivity in other marine populations by a high level of gene flow across 3,000 km coastline was reported in *S. longiceps* (Sukumaran *et al*. 2016), *Uca annulipes* (Silva *et al*. 2010) and *Neosarmatium meinerti* (Ragionieri *et al*. 2009). Thus, a lack of population genetic structure of *N. hexodon* in each Thailand coastline was plausibly maintained by a high dispersal ability in the larval phase and a short geographic distance. Also, genetic homogeneity among *N. hexodon* populations in each coastline could be the result of the panmixia of moving planktonic larva under the influence of the monsoon and the marine circulation patterns. In the Gulf of Thailand, the marine current is a clockwise gyre during the southwest monsoon and a counterclockwise gyre in the northeast monsoon period (Nakthon 1992), and in the Andaman Sea marine current always flows from the Malacca strait toward the Andaman Sea during the northeast and southeast monsoon (Sharp 1996). The lack of genetic structure was also reported in populations of other marine species in the Thailand coast such as the Gulf of Thailand; *Perna viridis* (Prakoon *et al*. 2010), *H. kuda* (Panithanarak *et al*. 2010), *A. pleuronectes* (Mahidol *et al*. 2007) and *Crassostrea belcheri* (Bussarawit & Simonsen 2006): the Andaman Sea; *Episesarma versicolor* (Supmee *et al*. 2012) and *P. monodon* (Supungul *et al*. 2000). From the genetic structure result in the study, we suggested that the implication to the management of the *N. hexodon* population in the Gulf of Thailand and the Andaman Sea should be managed as distinct units because each population carries a unique genetic structure. For example, to increase the abundance of *the N. hexodon* population in the Gulf of Thailand via restocking individuals should not be collected fish from the Andaman Sea because it could lead to outbreeding depression and genetic contamination of natural populations.

### Demographic History

In our study, all of the demographic history tests revealed that *N. hexodon* living in the Gulf of Thailand and the Andaman Sea had experienced population expansion. Firstly, the neutrality test showing negative Tajima’s *D* and Fu’s *Fs* deviated significantly (*p* < 0.05) from the neutral state. Tajima’s *D* test with a significant negative value might have been caused by slightly deleterious mutations, purifying selection, or a population expansion (Yang 2006). Also, Fu’s *Fs* statistics, a powerful statistical test for detecting demographic expansion from mitochondrial DNA sequence, indicated a significant negative value and population expansion (Ramirez-Soriano *et al*. 2008). Secondly, the demographic expansion was confirmed with the Bayesian skyline analysis that the expansion time of the *N. hexodon* population occurred around 10,000 years ago in the Gulf of Thailand and 4,000 years ago in the Andaman Sea during the Pleistocene glaciation period. The expansion time coincided with the violent climate changes that started in the same period. The estimated time of the population expansion obtained in this study supported the diversification of marine species in Southeast Asia from 2.4 million to 10,000 years ago (McMillan & Palumbi 1995). The Pleistocene isolation of the marine basin hypothesis revealed that the lowered sea levels during the Pleistocene ice ages affected the genetic structure of Indo-Pacific marine species between the Indian and the Pacific Ocean populations (McManus 1985). These two oceans were separated from each other during each glaciation as sea levels dropped up to 120 m below present-day levels and form land bridges between the Pacific and Indian Oceans (Voris 2000). Further, the land bridges prevented the dispersal of marine species between the oceans. Low dispersal over a long period would limit the gene flow of *N. hexodon* as evidence of a genetic structure in our study. Thus, the genetic diversity of *N. hexodon* may be clarified by the McManus hypothesis, which would have isolated the Gulf of Thailand and the Andaman Sea populations. Changes in sea levels during the Pleistocene period affected the diversification of other marine species living along the Thailand coast, such as *H. kuda* (Panithanarak *et al*. 2010), *A. pleuronectes* (Mahidol *et al*. 2007) and *Lutjanus russelli* (Klangnurak *et al*. 2012). In our study, the expansion time of the *N. hexodon* population in the Gulf of Thailand occurred around 10,000 years ago while in the Andaman Sea occurred around 4,000 years ago, indicating that the fish population in the Andaman Sea had a recent population expansion. Thirdly, the mismatch distribution was accepted by the sudden expansion model through the goodness of fit test. Fourthly, the estimated values of *θ*_1_ were higher than *θ*_0_ in every population indicating a demographic expansion. A minimum spanning network revealed a star-like topology in the Gulf of Thailand and the Andaman Sea group, indicating a signature of population expansion between these two seas (Slatkin & Hudson 1991).

## CONCLUSION

In conclusion, our study showed that the population genetic structure of *N. hexodon* living along the Thailand coast was separated into two populations: the Gulf of Thailand and the Andaman Sea. The cause of genetic structure is caused by a geographic barrier from the Thai-Malaysian peninsula and the Pleistocene isolation of the marine basin hypothesis. The results of demographic history indicated that the *N. hexodon* population had experienced population expansion during the Pleistocene glaciation period. The results suggested that *N. hexodon* population along the Thailand coast should be managed as two-unit programmes into the Gulf of Thailand and the Andaman Sea populations because each population carries a unique genetic structure.

## Figures and Tables

**Table 1:** Variation position among 18 mtDNA *COI* haplotypes of *N. hexodon* along the Thailand coast.

Haplotype	Nucleotide position													

	008	011	013	068	104	106	197	201	210	253	353	362	374	396
H01	T	A	C	G	G	G	C	G	C	T	A	A	T	T
H02	.	.	T	.	.	.	T	.	.	.	.	.	.	.
H03	.	.	T	.	.	A	T	.	.	.	.	.	.	.
H04	.	.	T	.	.	.	.	.	.	.	.	.	.	.
H05	.	.	T	.	.	.	T	.	.	C	.	.	.	.
H06	.	.	T	.	.	.	T	.	.	.	.	.	C	.
H07	.	.	T	.	A	.	T	.	.	.	.	G	.	.
H08	.	.	T	.	.	.	T	.	.	.	G	G	.	C
H09	.	.	T	.	A	.	T	.	.	.	G	G	.	.
H10	.	.	T	.	.	.	T	.	.	.	.	G	.	.
H11	.	.	T	.	.	.	T	.	.	.	G	G	.	.
H12	A	.	G	.	.	.	T	.	.	.	.	.	.	.
H13	.	.	T	A	.	.	T	.	.	.	.	G	.	.
H14	.	.	T	.	A	.	T	.	T	.	.	G	.	.
H15	.	.	T	.	.	.	T	.	T	.	.	G	.	.
H16	.	.	T	.	.	.	T	T	.	.	.	G	.	.
H17	.	T	T	.	A	.	T	.	.	.	.	G	.	.
H18	.	.	T	A	.	.	T	.	T	.	.	G	.	.

*Note*: All haplotypes are compared with haplotype 1. Dot (.) indicates identical nucleotides.

**Table 2:** Collecting localities, code of collecting site, number of individuals per sampling site (*N*), number of polymorphic sites, haplotype diversity (*h*) and nucleotide diversity (*π*) of *N. hexodon* along the Thailand coast.

Collecting localities	Code	*N*	No. polymorphic sites	No. haplotypes	Haplotype diversity (*h*) (mean ± SD)	Nucleotide diversity (*π*) (mean ± SD)
Chonburi	CH	16	5	6	0.742 ± 0.084	0.002 ± 0.000
Samut Songkhram	SM	15	3	5	0.705 ± 0.088	0.002 ± 0.000
Surat Thani	SR	16	3	5	0.608 ± 0.130	0.002 ± 0.000
Nakhon Si Thammarat	NS	14	2	3	0.582 ± 0.092	0.001 ± 0.000
Songkhla	SK	16	6	7	0.792 ± 0.089	0.003 ± 0.000

Gulf of Thailand		**77**	**9**	**12**	**0.740** ± **0.035**	**0.002** ± **0.000**

Satun	ST	17	3	4	0.500 ± 0.135	0.001 ± 0.000
Trang	TG	18	4	5	0.614 ± 0.117	0.001 ± 0.000
Krabi	KB	15	3	4	0.543 ± 0.133	0.001 ± 0.000
Phang Nga	PN	15	1	2	0.133 ± 0.112	0.000 ± 0.000

Andaman Sea		**65**	**5**	**6**	**0.468** ± **0.074**	**0.001** ± **0.000**

**Table 3:** Haplotype distributions of *N. hexodon* from 9 localities along the Thailand coast.

Haplotype	CH	SM	SR	NS	SK	ST	TG	KB	PN	Total
H01	-	-	-	-	-	2	-	-	-	2
H02	-	-	-	-	3	12	11	10	14	50
H03	-	-	-	-	-	2	2	-	-	4
H04	-	-	-	-	-	1	1	3	-	5
H05	-	-	-	-	-	-	1	1	1	3
H06	-	-	-	-	-	-	3	1	-	4
H07	5	1	10	8	7	-	-	-	-	31
H08	-	-	1	-	1	-	-	-	-	2
H09	-	-	2	-	1	-	-	-	-	3
H10	7	7	2	5	2	-	-	-	-	23
H11	-	-	1	1	1	-	-	-	-	3
H12	-	-	-	-	1	-	-	-	-	1
H13	-	5	-	-	-	-	-	-	-	5
H14	-	1	-	-	-	-	-	-	-	1
H15	1	1	-	-	-	-	-	-	-	2
H16	1	-	-	-	-	-	-	-	-	1
H17	1	-	-	-	-	-	-	-	-	1
H18	1	-	-	-	-	-	-	-	-	1

Total	16	15	16	14	16	17	18	15	15	142

*Note*: Station codes are given in Table 2.

**Table 4:** Hierarchical AMOVA of *N. hexodon.*

Source of variation	df	Sum of squares	Variance components	Percentage of variation	*p*-value
**Single region**					
Among populations	8	48.772	0.359Va	46.08	*Φ_ST_* = 0.460*(*p* = 0.000)
Within populations	133	56.024	0.421Vb	53.92	

Total	141	104.796	0.781		

**Gulf of Thailand and Andaman Sea**					
Among groups	1	40.891	0.563Va	54.76	*Φ_CT_* = 0.547*(*p* = 0.008)
Among populations within groups	7	7.881	0.044Vb	4.35	*Φ_SC_* = 0.096*(*p* = 0.000)
Among populations	133	56.024	0.421Vc	40.90	*Φ_ST_* = 0.591*(*p* = 0.000)

Total	141	104.796	1.029		

**Lower and upper Gulf of Thailand**					
Among groups	1	4.070	0.086Va	13.37	*Φ_CT_* = 0.133 (*p* = 0.098)
Among populations within groups	3	2.623	0.021Vb	3.41	*Φ_SC_* = 0.030 (*p* = 0.094)
Among populations	72	38.657	0.536Vc	83.23	*Φ_ST_* = 0.167 (*p* = 0.090)

Total	76	45.351	0.645		

**Lower and upper Andaman Sea**					
Among groups	1	0.335	−0.002Va	−0.97	*Φ_CT_* = −0.009 (*p* = 0.672)
Among populations within groups	2	0.852	0.008Vb	3.00	*Φ_SC_* = 0.029 (*p* = 0.147)
Among populations	61	17.367	0.284Vc	97.97	*Φ_ST_* = 0.020 (*p* = 0.145)

Total	64	18.554	0.290		

*Note*:

* significant differentiation (*p* < 0.05).

**Table 5:** Population pairwise *F_ST_* values of *N. hexodon*.

CH		Gulf of Thailand				Andaman Sea				
	
		SM	SR	NS	SK	ST	TG	KB	PN	
Gulf of Thailand	CH	–								
	SM	0.064	–							
	SR	0.143*	0.351*	–						
	NS	0.011	0.245*	0.016	–					
	SK	0.046	0.179*	0.042	0.011	–				
Andaman Sea	ST	0.559*	0.574*	0.662*	0.652*	0.416*	–			
	TG	0.561*	0.577*	0.655*	0.655*	0.421*	0.022	–		
	KB	0.572*	0.591*	0.678*	0.676*	0.422*	−0.008	0.003	–	
	PN	0.639*	0.668*	0.746*	0.796*	0.496*	0.069	0.032	0.050	–

*Note*:

* significant differentiation (*p* < 0.05).

Stations codes are given in Table 2.

**Table 6:** Parameter indices of mismatch distribution analysis and neutrality test of *N. hexodon*.

Collecting localities	Tajima’s *D*	Fu’s *Fs*	SSD^a^	rg^b^	*θ_0_*^c^	*θ_1_*^d^
Chonburi	−0.576	−2.360*	0.011	0.126	0.000	99999.000
Samut Songkhram	−0.157	−1.598	0.017	0.152	0.000	99999.000
Surat Thani	−0.067	−1.625	0.001	0.062	0.000	99999.000
Nakhon Si Thammarat	−0.178	−0.055	0.021	0.178	0.000	99999.000
Songkhla	−0.331	−2.356*	0.007	0.053	0.000	16.235
Gulf of Thailand	−1.034*	−2.479*	0.001	0.109	0.000	99999.000
Satun	−0.438	−0.827	0.003	0.083	0.000	11.247
Trang	−1.128	−2.095	0.022	0.179	0.000	99999.000
Krabi	−1.009	−1.419	0.019	0.172	0.000	99999.000
Phang Nga	−1.159	−0.648	0.037	0.555	0.112	12.045
Andaman Sea	−0.941*	−5.918*	0.005	0.092	0.002	99999.000

*Notes*:

* significant differentiation (*p* < 0.05),

a sum of squared deviations,

b raggedness index,

c population size before expansion (*θ_0_* = 2N_0_μ),

d population size after expansion (*θ_1_* = 2N_1_μ).
